# Supplementary material for: Exploring penetrance of clinically relevant variants in over 800,000 humans from the Genome Aggregation Database
Source: Nat Commun. 2025 Oct 31;16:9623. doi: 10.1038/s41467-025-61698-x (PMC12579199; doi:10.1038/s41467-025-61698-x)
Supplement: Supplementary file 1 — Supplementary Information [file 41467_2025_61698_MOESM1_ESM.pdf]

Supplementary Information

Exploring disease-variants and variable penetrance in over 800,000 individuals from the Genome Aggregation Database

Sanna Gudmundsson[1,2,3,4*], Moriel Singer-Berk[1], Sarah L. Stenton[1,2,3], Julia K. Goodrich[1], Michael W. Wilson[1], Jonah Einson[5], Nicholas A Watts[1], Genome Aggregation Database Consortium, Tuuli Lappalainen[4,5], Heidi L. Rehm[1,2], Daniel G. MacArthur[1,6,7], Anne O'Donnell-Luria[1,2,3*]

Content

Supplementary Figures page 2-14

Supplementary Data page 15

Supplementary Note page 16

Genome Aggregation Database Consortium Author Information page 17

Funding page 17

Competing Interests page 22-23

Supplementary Figures

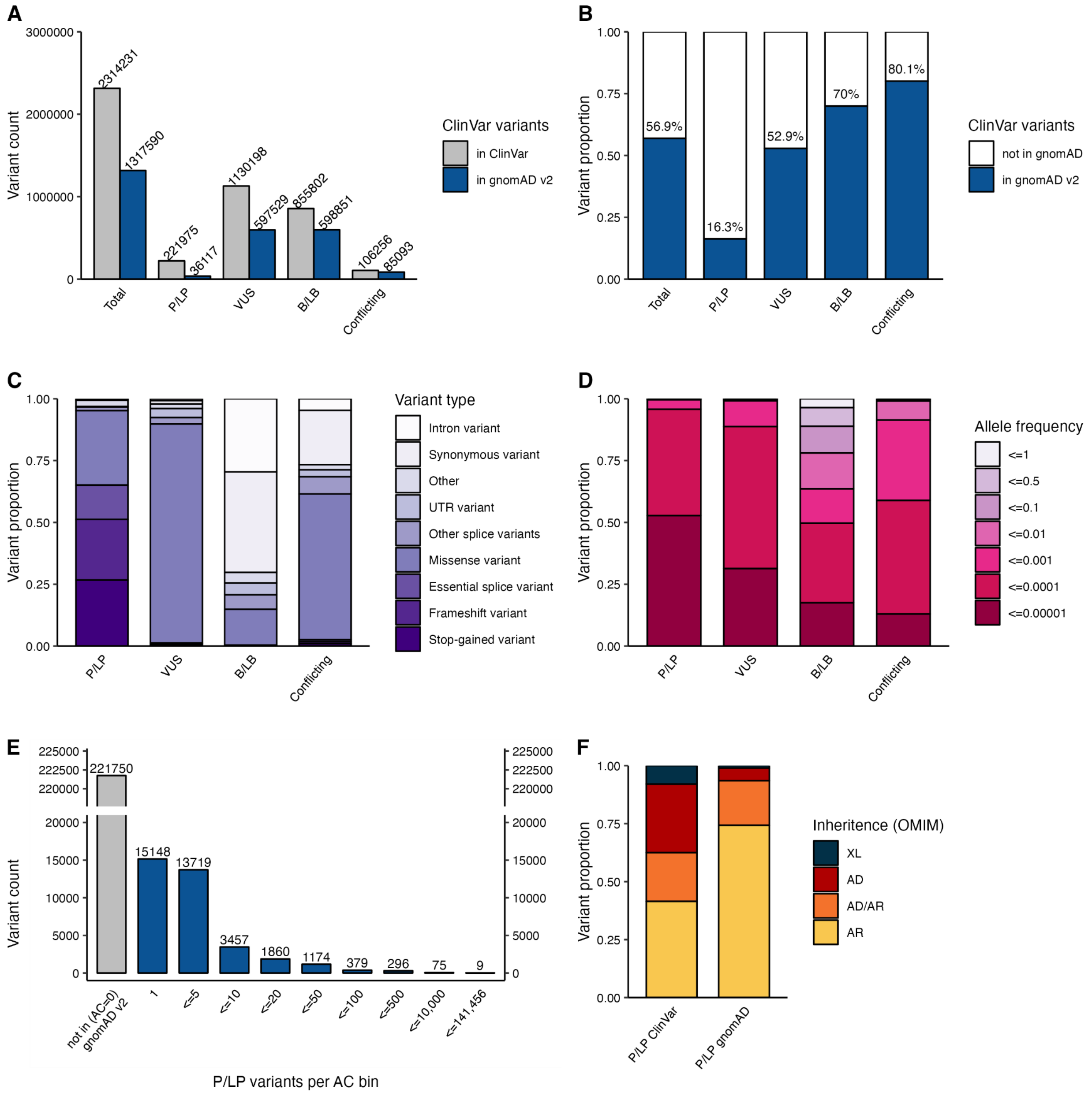

Fig. S1: Representation of ClinVar variants in 141,456 individuals in gnomAD v2. (A) Variant count of ClinVar variants in ClinVar (grey) vs. gnomAD (blue) in each classification category pathogenic/likely pathogenic (P/LP), variant of uncertain significance (VUS), benign/likely benign (B/LB) or with conflicting classifications. (B) Percentage of ClinVar variants reported in gnomAD in at least one individual. (C) The proportion of variants by variant type within each

clinical significance classification and, (D) within each allele frequency bin. (E) Total number of P/LP variants within each allele count bin. (F) The inheritance pattern of the gene harboring the P/LP variants in gnomAD vs. inheritance of all variants in ClinVar.

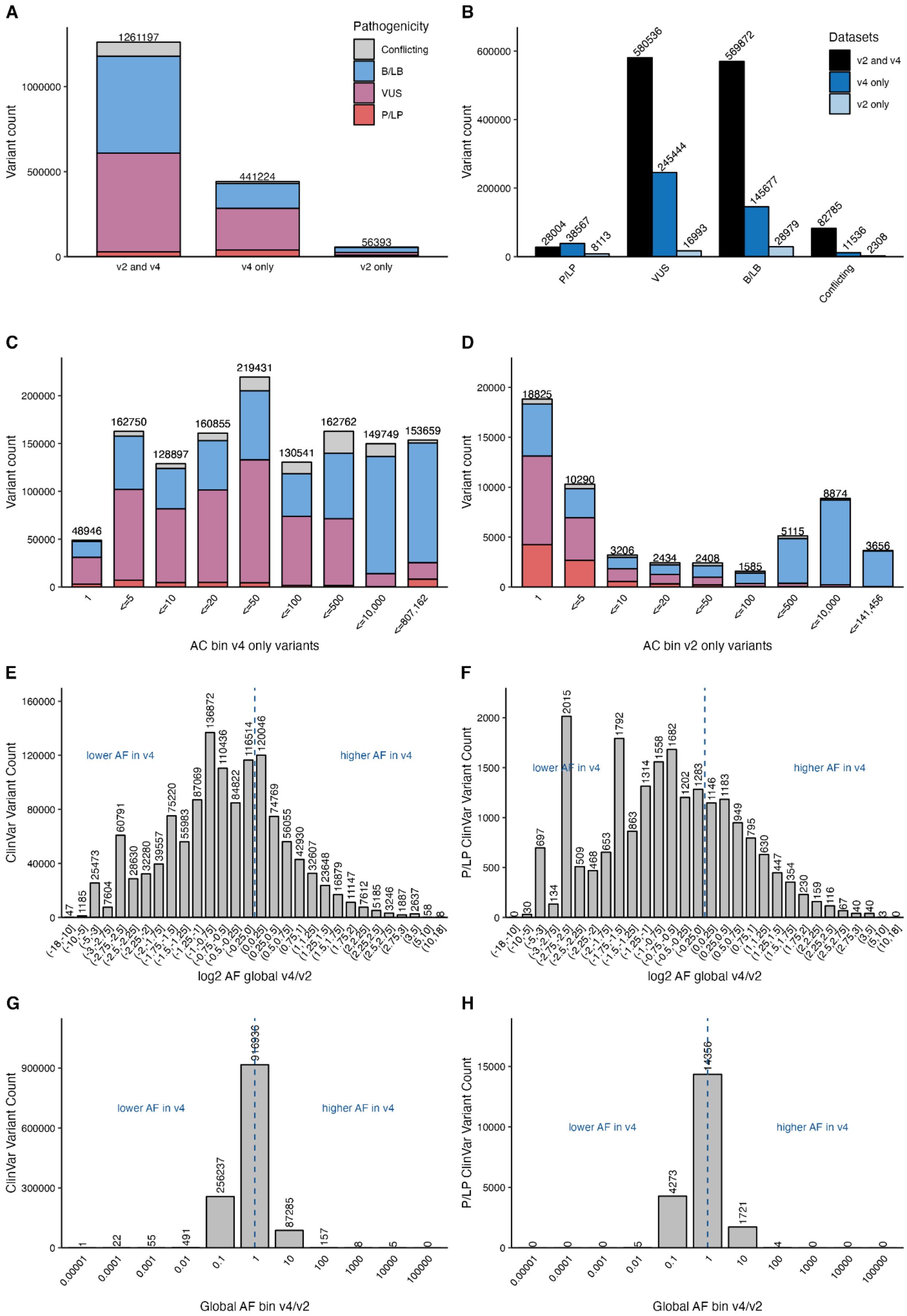

A
Pathogenicity
Conflicting
B/LB
VUS
P/LP
Variant count
1261197
441224
56393
v2 and v4
v4 only
v2 only
B
Datasets
v2 and v4
v4 only
v2 only
Variant count
28004
38567
8113
580536
245444
16993
569872
145677
28979
82785
11536
2308
P/LP
VUS
B/LB
Conflicting
C
Variant count
48946
162750
128897
160855
219431
130541
162762
149749
153659
1
<=5
<=10
<=20
<=50
<=100
<=500
<=10,000
<=807,162
AC bin v4 only variants
D
Variant count
18825
10290
3206
2434
2408
1585
5115
8874
3656
1
<=5
<=10
<=20
<=50
<=100
<=500
<=10,000
<=141,456
AC bin v2 only variants
E
ClinVar Variant Count
lower AF in v4
higher AF in v4
47
1185
25473
7604
60791
28630
32280
39557
75220
55983
87069
136872
110436
84822
116514
120046
74769
56055
42930
32607
23648
16879
11147
7612
5185
3246
1887
2637
58
8
log2 AF global v4/v2
F
P/LP ClinVar Variant Count
lower AF in v4
higher AF in v4
0
30
697
134
2015
509
468
653
1792
863
1314
1558
1682
1202
1283
1146
1183
949
795
630
447
354
230
159
116
67
40
40
3
0
log2 AF global v4/v2
G
ClinVar Variant Count
lower AF in v4
higher AF in v4
1
22
55
491
256237
916936
87285
157
8
5
0
0.00001
0.0001
0.001
0.01
0.1
1
10
100
1000
10000
100000
Global AF bin v4/v2
H
P/LP ClinVar Variant Count
lower AF in v4
higher AF in v4
0
0
0
5
4273
14356
1721
4
0
0
0
0.00001
0.0001
0.001
0.01
0.1
1
10
100
1000
10000
100000
Global AF bin v4/v2

Fig. S2: ClinVar variants represented in gnomAD v2 and v4. (A-B) Total ClinVar variant count per dataset grouped by pathogenicity. (C-D) Allele count (AC) bins for variants only present in v4 (C) or v2 (D). (E-F) Allele frequency (AF) ratio v4 compared to v2 (log2 scale) all ClinVar variants (E) and ClinVar P/LP variants (F) that are present in both v2 and v4 datasets. (G-H) The ratio between AF bins in v4 compared to v2 all ClinVar variants (G) and ClinVar P/LP variants (H) that are present in both v2 and v4 datasets. The majority of variants belong to the same AF bin in both datasets, while 21% (n=4278) of P/LP variants are 10 times less common (equal to or below 0.1) in v4.

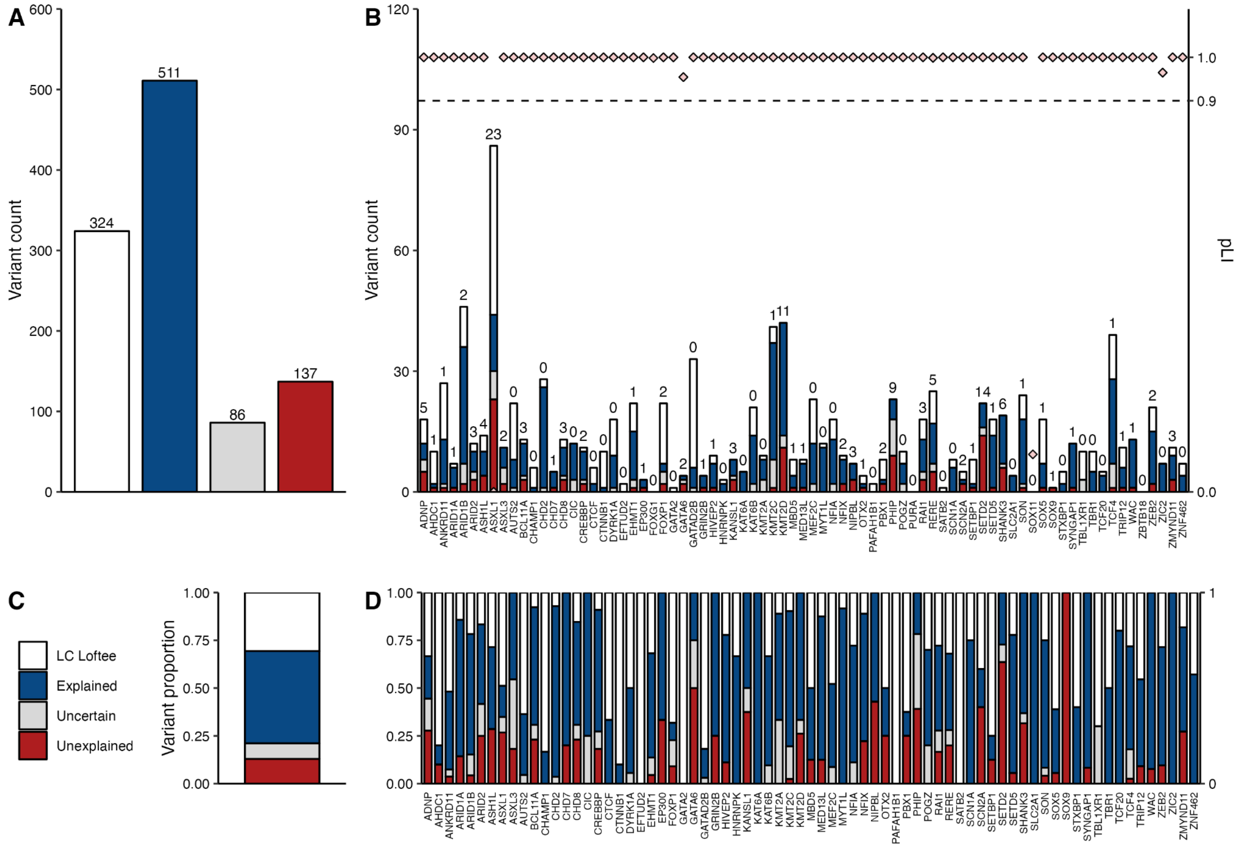

Fig. S3: Investigation of all pLoF variants (n=1,058) in 77 genes associated with severe, early-onset highly penetrance disease. Loss-Of-Function Transcript Effect Estimator (LOFTEE) filtered 30.6% (324/1,058) of pLoF variants as low-confidence (LC; white). Application of the Singer-Berk et al., pLoF framework led to 48.3% (511/1,058) assessed as likely not/not pLoF and thus considered explained (blue), and 8.1% (86/1,058) excluded as uncertain (grey). Of 1058 pLoF variants, 12,9% (n=137) the lack of disease manifestation in gnomAD individuals was still unexplained (red) and subject to further evaluation. (A) Variant counts per category across all variants, (B) by gene, and (C) all variants displayed as proportion (D) per gene.

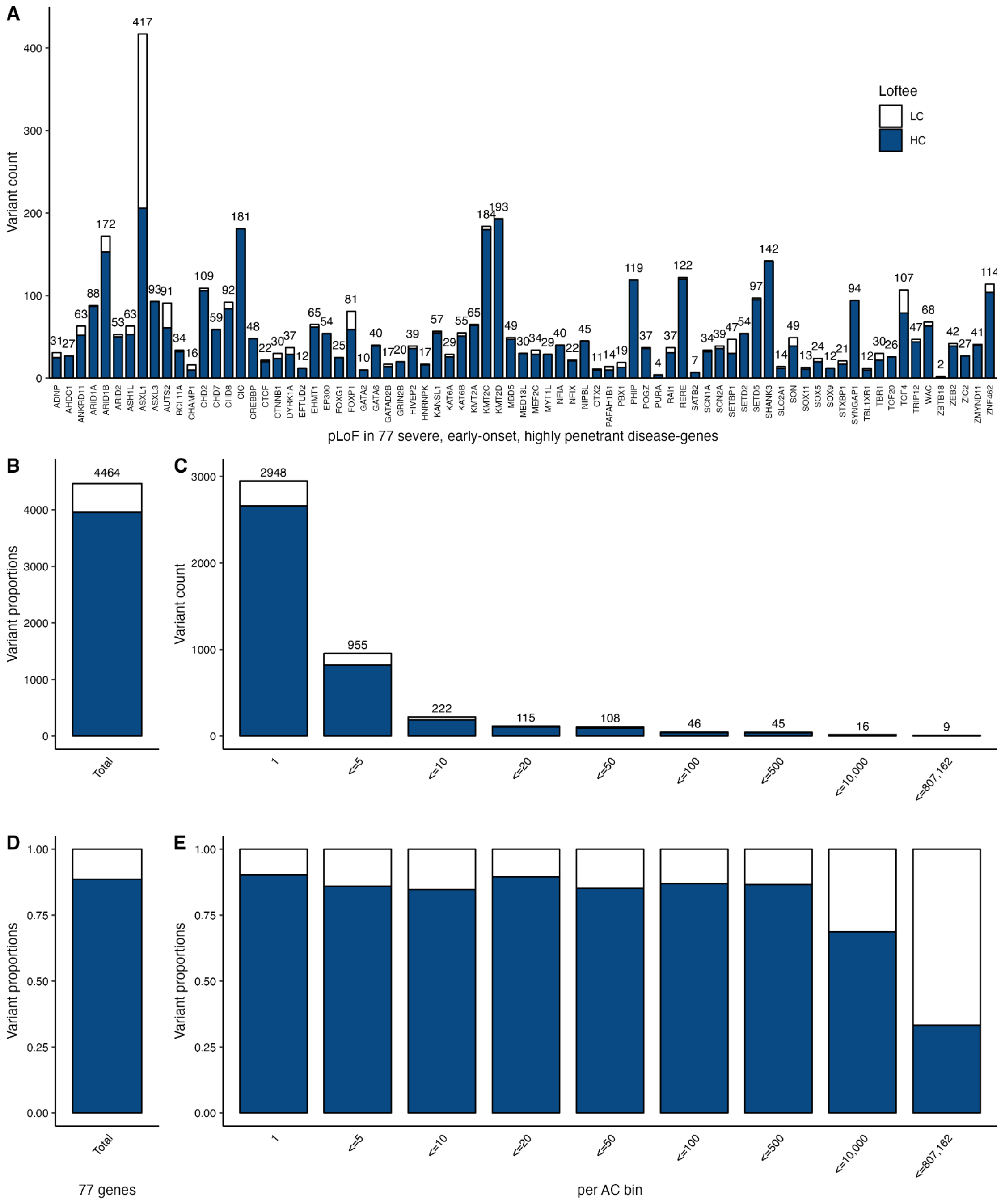

Fig. S4: pLoF variants in the 77 genes in 807,162 individuals in gnomAD v4. (A) Variant count per gene, colored by low confidence (LC, white) or high-confidence (HC, blue) by Loss-Of-Function Transcript Effect Estimator (LOFTEE). (B) The total number of variants and (C) variants per allele count (AC) bin. (D-E) Proportion of LC and HC variants for all variants (D), per AC bin (E).

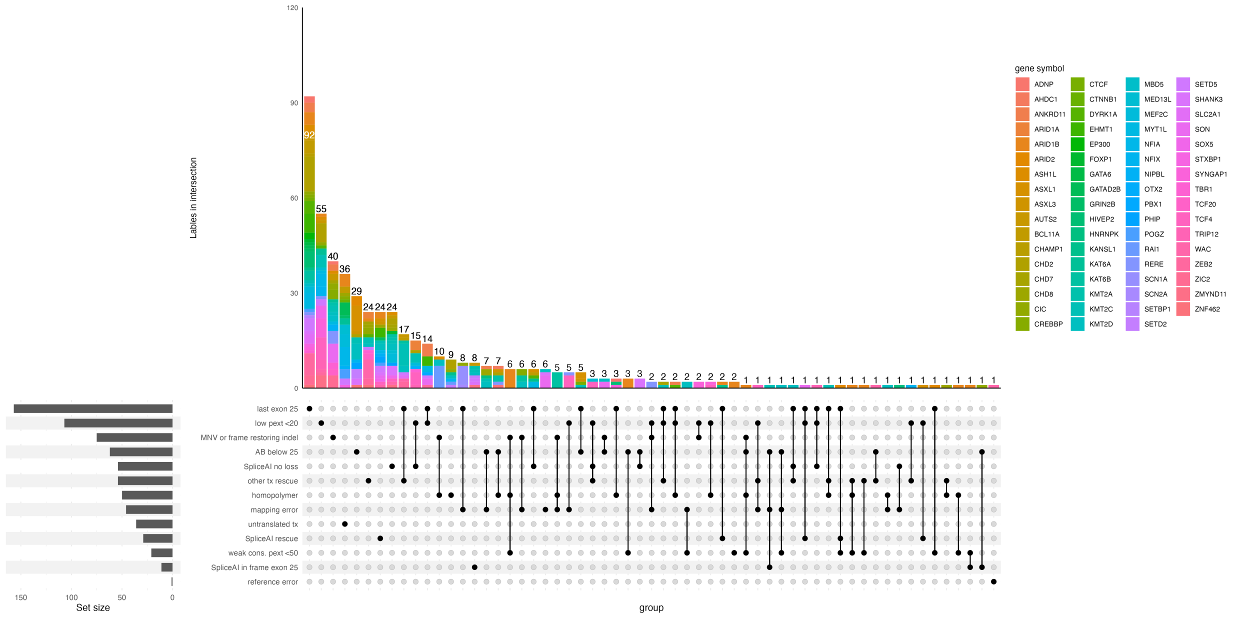

Fig. S5: Upset plot including reasons for variants labeled as not/likely not LoF, colored by gene.

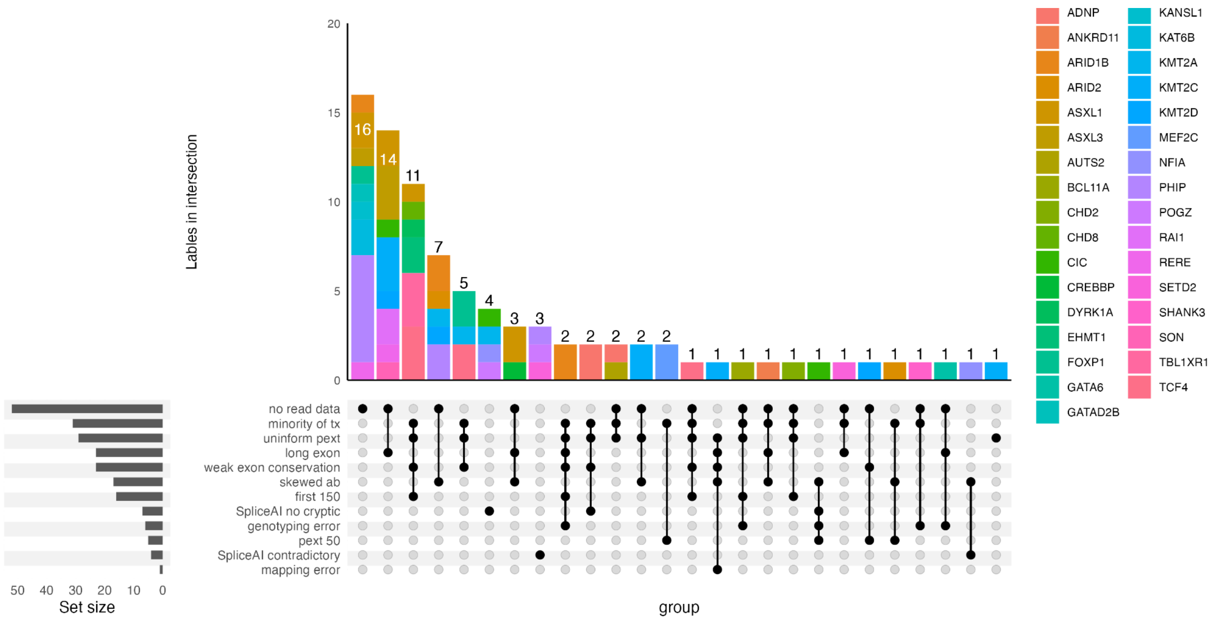

Fig. S6: Upsetplot for variants labeled as uncertain, colored by gene.

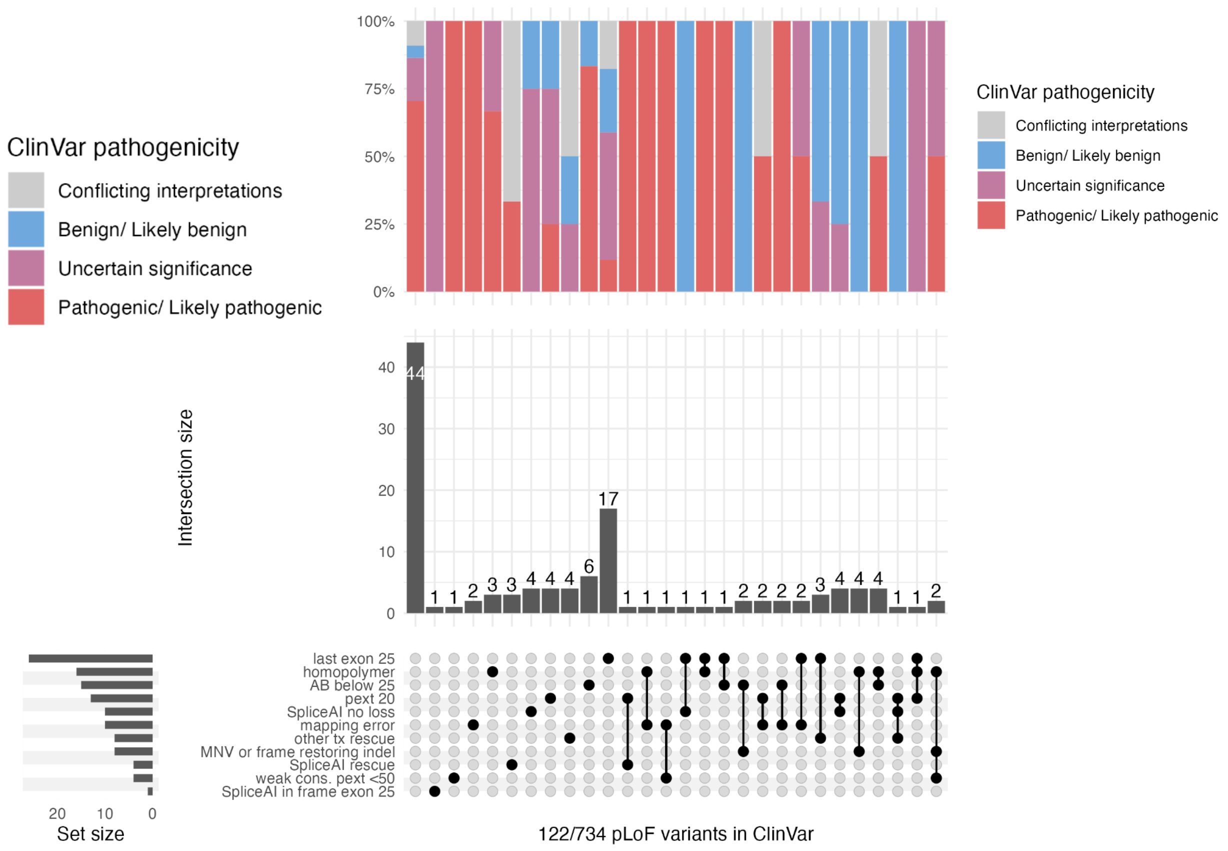

Fig. S7: Upsetplot of 122 of 734 pLoF also reported in ClinVar including labels that result in a not/likely not pLoF verdict listed, colored by ClinVar pathogenicity category. The 44 variants (first bar) with no flags had a verdict of “LoF”, “Likely LoF” or “Uncertain”.

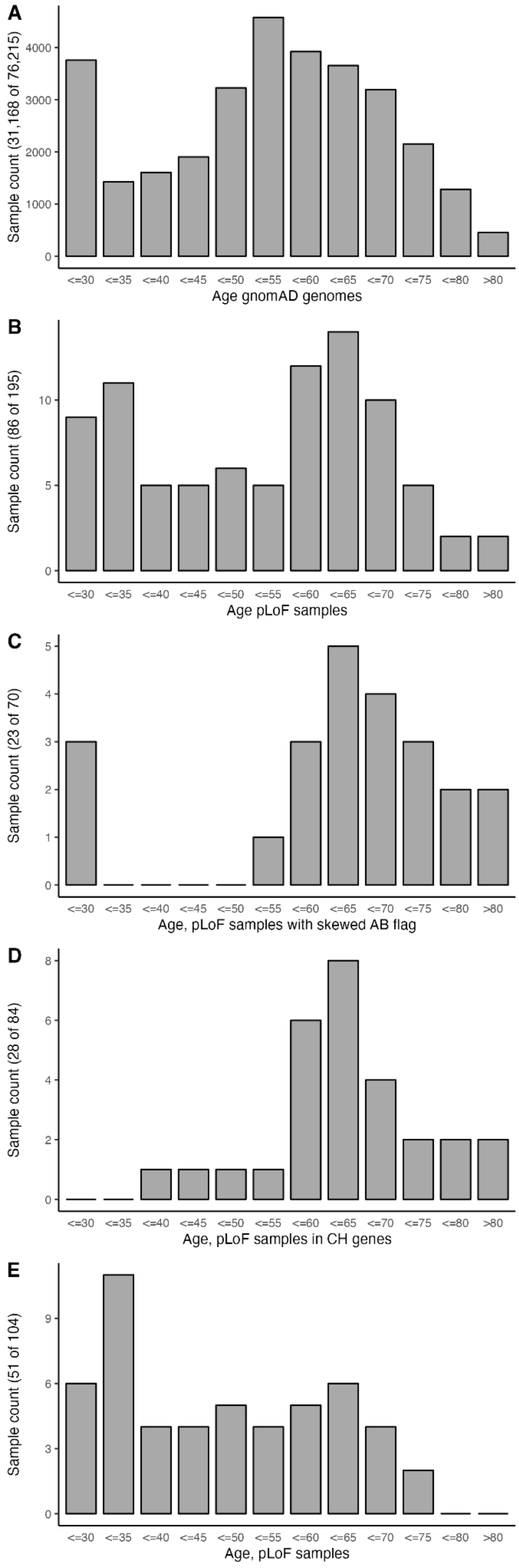

Fig. S8: Skewed age and allele balance (AB) as an indicator of somatic variation. (A) Age distribution in gnomAD genomes, aga data available for 31,168 of 76,215 individuals. (B) Age distribution in 195 samples with 104 pLoF variants passing LoF curation and excluding pLoF in samples from the 1000 Genomes Project, age data available for 86 of 195 samples. (C) Age distribution in 70 samples where the pLoF shows skewed AB ratio (alternative allele >25% but <35%), age data available for 23 of 70 samples. Skewed towards elderly people compared to gnomAD genomes (two-sided Wilcoxon rank sum test, $p = 0.0013$) (D) Age distribution in 84 samples with pLoF variant in genes associated with clonal hematopoiesis (CH), age data available for 28 of 84 samples. Skewed towards elderly people compared to gnomAD genomes (two-sided Wilcoxon rank sum test, $p = 0.00027$) (E) Age distribution of remaining 104 samples with 50 pLoF variants passing somatic filtering, age data available for 51 of 104 samples.

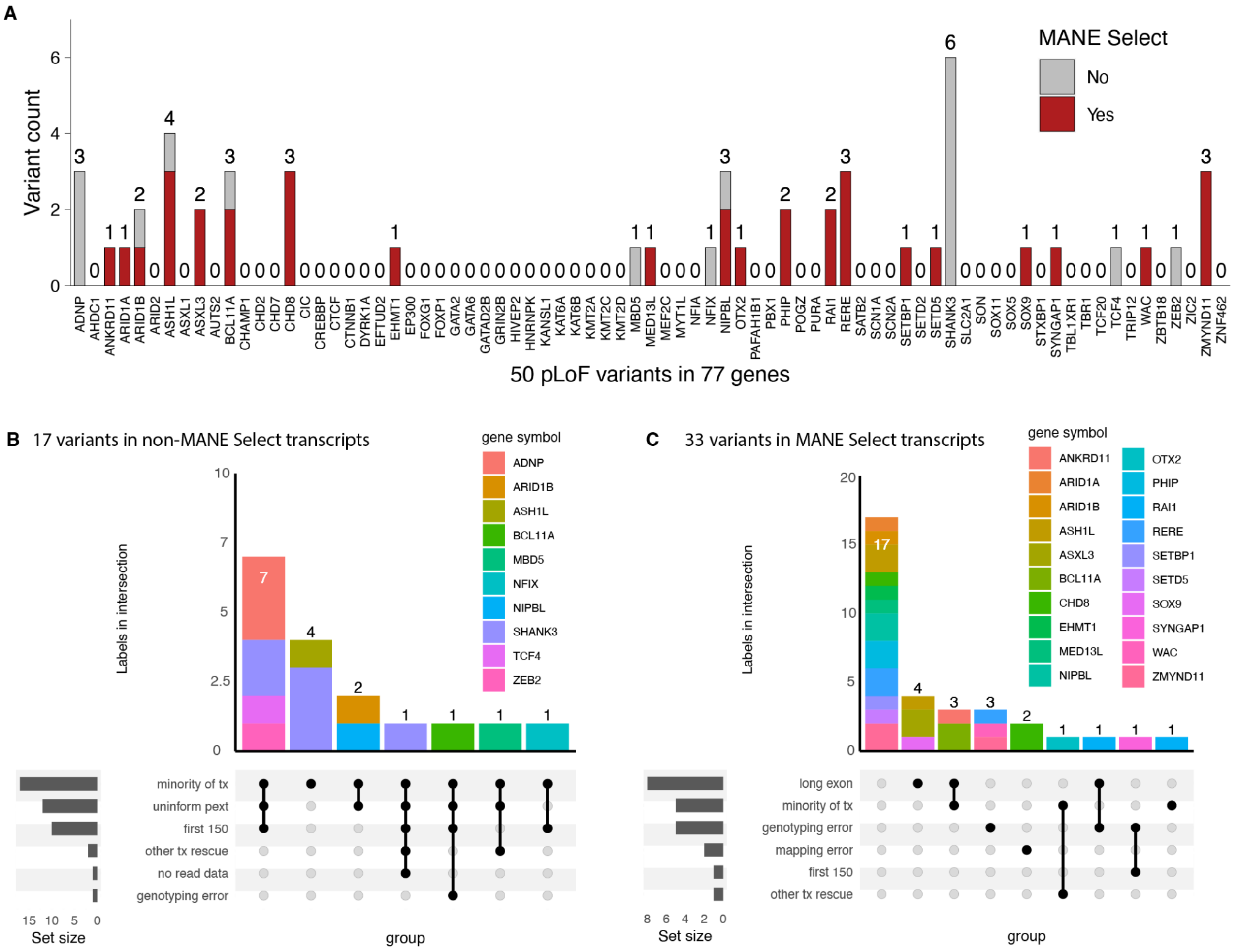

Fig. S9: Of 734 pLoF variants in 77 genes associated with haploinsufficiency disease, only 50 pLoF variants (6.8%) remained unexplained after careful scrutinization (A) Variant count per gene colored by whether the variant is present in the MANE Select transcript (red) or not (grey). (B) Upset plot of features of 17 variants in non-MANE Select transcripts and (C) 33 pLoF variants in MANE Select transcripts.

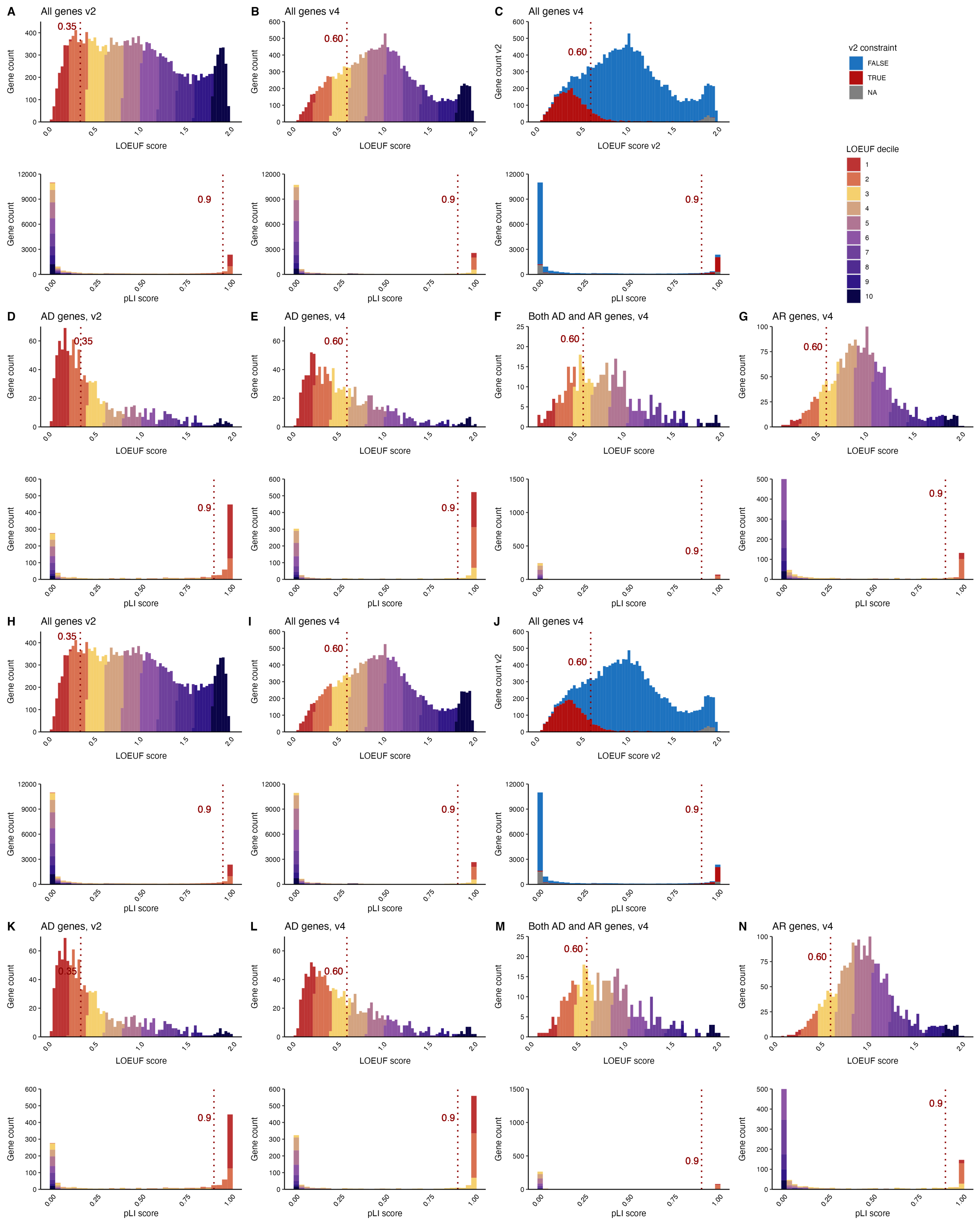

Fig. S10: Constraint scores, LOEUF score (upper) and pLI (lower), colored by LOEUF decile

(A) for all genes in v2, (B) all genes in v4, (C) constraint scores in v4 colored by constraint in v2

(LOEUF ≤ 0.35, pLI≥ 0.9, red) and not constraint in v2 (LOEUF >0.35, pLI <0.9, blue). (D) Distribution of constraint scores for genes associated with diseases of autosomal dominant (AD) in v2 (D) and v4 (E), as well as for v4 both AD and autosomal recessive (AR), and (F) AR inheritance in OMIM (G). Transcripts for v4 match transcripts of v2. Dotted line indicates threshold for loss-of-function constraint (0.35 in v2 and 0.60 in v4 for LOEUF, 0.9 for pLI). (H)-(N) Replication of A-G using MANE Select transcripts in v4 (rather than matching v4 transcripts to v2 in A-G).

Supplementary Data

Supplementary Data S1: Allele count for allele frequency (AF) bins, Fig. 1d.

Supplementary Data S2: Allele count for P/LP allele count (AC) bins, Fig. 1e.

Supplementary Data S3: Assessment of 90 unique P/LP occurring in combination with pLoF when investigating local pLoF as rescue of 3957 P/LP in ClinVar variants.

Supplementary Data S4: 77 haploinsufficiency genes associated with severe disorders not expected to be compatible with participation in common disease studies (e.g., gnomAD), early onset (before the age of three), and reported as highly penetrant (using *de novo* rate as a proxy for penetrance).

Supplementary Data S5: The specific set of rules used for this project modified for conservative curation using the advanced framework for loss-of-function curation previously published by this group[25]**.**

Supplementary Data S6: Full curation results of 734 pLoF variants found in 77 haploinsufficiency genes in gnomAD v4 genomes.

Supplementary Data S7: Counts for each ClinVar classification and pLoF curation category, 108 of 734 pLoF variants were reported as benign, likely benign, unkown significance, likely pathogenic or pathogenic. Additional 14 variants were reported but excluded due to conflicting interpretations (all 122 variants are included in Figure 3g).

Supplementary Data S8: sQTL results from all pLoF variants.

Supplementary Data S9: Genotypes for sQTL in individuals with MEF2C pLoF variants.

Supplementary Note

Rescue by local pLoF events in a subset of P/LP in ClinVar

Of 3957 of P/LP variants (found in in 31,014 of 807,162 individuals in gnomAD), 90 unique P/LP variants in 222 individuals occurred in combination with a pLoF (AF ≤ 0.01, depth < 10, genotype quality < 20, minor allele balance < 0.2) in the same gene. Evaluation following three steps revealed one interesting case: a pLoF in combination with an otherwise lethal autosomal dominant variant in *GJB2* (see results). Additional to the highlighted GJB2 case (results), one P/LP variant (11-121166704-G-A) in *TECTA* associated with autosomal non-syndromic dominant hearing loss reportedly acting through dominant negative effect[39] was rescued by an MNV variant where the secondary 11-121166705-C-A variant occurring in the same codon as the P/LP variant altered the missense variant to a nonsense variant in one individual. Biallelic nonsense variants in *TECTA* are associated with autosomal recessive hearing loss, and thus this heterozygous MNV is not disease-causing in this one individual. We also found one combination of a P/LP variant (7-87453031-C-T) in *ABCB4* gene reported to cause Intrahepatic cholestasis of pregnancy in combination with a pLoF splice variant in 17 individuals of European ancestry. The other 87 unique P/LP variants occurring in combination with a pLoF were excluded based on (1) carriership of a pLoF variant was not found in the majority (≤50%) of individuals with the P/LP variant (79 variants), (2) the pLoF variant showed evidence of being “not/likely not pLoF” and thus not resulting in ablated protein product (6 variants), and (3) the P/LP variant was not acting through dominant gain-of-function mechanism (2 variant) (Supplementary Data S3).

Genome Aggregation Database Consortium Author Information

Funding

Elizabeth G. Atkinson: National Institutes of Mental Health (K01MH121659), the Caroline Wiess Law Fund for Research in Molecular Medicine, and the ARCO Foundation Young Teacher-Investigator Fund at Baylor College of Medicine

Emelia J. Benjamin: R01HL092577; American Heart Association AF AHA_18SFRN34110082

Matthew J. Bown: British Heart Foundation awards CS/14/2/30841 and RG/18/10/33842

Steven Brant: National Institutes of Health DK062431

Lea A. Chen: NIH, New York Crohn's Foundation, Crohn's and Colitis Foundation

Richard H. Duerr: U01 DK062420

Ravindranath Duggirala: U01 DK085524 National Institute for Diabetes and Digestive and Kidney Diseases (NIDDK)

Josée Dupuis: National Institute for Diabetes and Digestive and Kidney Diseases (NIDDK) R DK

Roberto Elosua: Agència de Gestió d'Ajuts Universitaris i de Recerca: 2021 SGR 00144

Jeanette Erdmann: VIAgenomics, Leducq network PlaqOmics, Deutsche Forschungsgemeinschaft Cluster of Excellence “Precision Medicine in Chronic Inflammation” (EXC2167);

Martti Färkkilä: State funding for university level health research

Laura D. Gauthier: Intel, Illumina

Benjamin Glaser: 5U01 DK085584

Stephen J. Glatt: U.S. NIMH Grant R MH

Leif Groop: The Academy of Finland and University of Helsinki: Center of Excellence for Complex Disease Genetics (grant number 312063 and 336822), Sigrid Jusélius Foundation; IMI 2 (grant No 115974 and 15881)

Christopher Haiman: U01CA164973

Mikko Hiltunen: Academy of Finland (grant 338182, 353053), Sigrid Jusélius Foundation, the Strategic Neuroscience Funding of the University of Eastern Finland

Chaim Jalas: Bonei Olam

Mikko Kallela: Grants from State funding for university level health research and from Department of Neurology, Helsinki University, Central Hospital; Grant from Maire Taponen Foundation

Jaakko Kaprio: Academy of Finland (grant 352792)

Mikael Landén: Swedish Medical Research Council (2022-00642)

Terho Lehtimäki: Academy of Finland (grant 356405), VTR Pirha

Ruth J.F. Loos: Novo Nordisk Foundation (NNF18CC0034900, NNF20OC0059313); NIH (R01DK110113; R01DK124097)

Ronald C.W. Ma: Research Grants Council of the Hong Kong Special Administrative Region (CU R4012-18), Research Grants; Council Theme-based Research Scheme (T12-402/13N), University Grants Committee Research Grants Matching Scheme

Jaume Marrugat: Agència de Gestió d'Ajuts Universitaris i de Recerca: 2021 SGR 00144

Jacob L. McCauley: National Institute of Diabetes and Digestive and Kidney Disease Grant R01DK104844

Michael C. O'Donovan: Medical Research Council UK: Centre Grant No. MR/L010305/1, Program Grant No. G0800509

Yukinori Okada: JSPS KAKENHI (19H01021, 20K21834), AMED (JP21km0405211, JP21ek0109413, JP21gm4010006, JP21km0405217, JP21ek0410075), JST Moonshot R&D (JPMJMS2021)

Michael J. Owen: Medical Research Council UK: Centre Grant No. MR/L010305/1, Program Grant No. G0800509

Aarno Palotie: the Academy of Finland Center of Excellence for Complex Disease Genetics (grant numbers 312074 and 336824) and Sigrid Jusélius Foundation

Heidi Rehm: U24HG011450

John D. Rioux: National Institute of Diabetes and Digestive and Kidney Diseases (NIDDK; DK062432), from the Canadian Institutes of Health (CIHR GPG 102170), from Genome Canada/Génome Québec (GPH-129341), and a Canada Research Chair (#230625)

Samuli Ripatti: the Academy of Finland Center of Excellence for Complex Disease Genetics (grant number) Sigrid Jusélius Foundation

Jerome I. Rotter: Trans-Omics in Precision Medicine (TOPMed) program was supported by the National Heart, Lung and Blood Institute (NHLBI). WGS for “NHLBI TOPMed: Multi-Ethnic Study of Atherosclerosis (MESA)” (phs001416.v1.p1) was performed at the Broad Institute of MIT and Harvard (3U54HG003067-13S1). Core support including centralized genomic read mapping and genotype calling, along with variant quality metrics and filtering were provided by the TOPMed Informatics Research Center (3R01HL-117626-02S1; contract HHSN268201800002I). Core support including phenotype harmonization, data management, sample-identity QC, and general program coordination were provided by the TOPMed Data Coordinating Center (R01HL-120393; U01HL-120393; contract HHSN268201800001I). We gratefully acknowledge the studies and participants who provided biological samples and data for MESA and TOPMed. JSK was supported by the Pulmonary Fibrosis Foundation Scholars Award and grant K23-HL-150301 from the NHLBI. MRA was supported by grant K23-HL-150280, AJP was supported by grant K23-HL-140199, and AM was supported by R01-HL131565 from the NHLBI. EJB was supported by grant K23-AR-075112 from the National Institute of Arthritis and Musculoskeletal and Skin Diseases.The MESA project is conducted and supported by the National Heart, Lung, and Blood Institute (NHLBI) in collaboration with MESA investigators. Support for MESA is provided by contracts 75N92020D00001, HHSN268201500003I, N01-HC-95159, 75N92020D00005, N01-HC-95160, 75N92020D00002, N01-HC-95161, 75N92020D00003, N01-HC-95162, 75N92020D00006, N01-HC-95163, 75N92020D00004, N01-HC-95164, 75N92020D00007, N01-HC-95165, N01-HC-95166, N01-HC-95167, N01-HC-95168, N01-HC-

95169, UL1-TR-000040, UL1-TR-001079, and UL1-TR-001420. Also supported in part by the National Center for Advancing Translational Sciences, CTSI grant UL1TR001881, and the National Institute of Diabetes and Digestive and Kidney Disease Diabetes Research Center (DRC) grant DK063491 to the Southern California Diabetes Endocrinology Research Center

Veikko Salomaa: Juho Vainio Foundation and Finnish Foundation for Cardiovascular Research

Jeremiah Scharf: NIH Grants U01 NS40024, K02 NS085048, NS102371

Eleanor G. Seaby: Kerkut Charitable Trust, Foulkes Fellowship, University of Southampton Presidential Scholarship

Edwin K. Silverman: NIH Grants U01 HL089856 and U01 HL089897

J. Gustav Smith: The Swedish Heart-Lung Foundation (2022-0344, 2022-0345), the Swedish Research Council (2021-02273), the European Research Council (ERC-STG-2015-679242), Gothenburg University, Skåne University Hospital, governmental funding of clinical research within the Swedish National Health Service, a generous donation from the Knut and Alice Wallenberg foundation to the Wallenberg Center for Molecular Medicine in Lund, and funding from the Swedish Research Council (Linnaeus grant Dnr 349-2006-237, Strategic Research Area Exodiab Dnr 2009-1039) and Swedish Foundation for Strategic Research (Dnr IRC15-0067) to the Lund University Diabetes Center

Harry Sokol: AgroParisTech, Jouy en Josas, France

Nathan O. Stitziel: National Human Genome Reserach Institute Grant UM1HG008853

Kent D. Taylor: Trans-Omics in Precision Medicine (TOPMed) program was supported by the National Heart, Lung and Blood Institute (NHLBI). WGS for “NHLBI TOPMed: Multi-Ethnic Study of Atherosclerosis (MESA)” (phs001416.v1.p1) was performed at the Broad Institute of MIT and Harvard (3U54HG003067-13S1). Core support including centralized genomic read mapping and genotype calling, along with variant quality metrics and filtering were provided by the TOPMed Informatics Research Center (3R01HL-117626-02S1; contract HHSN268201800002I). Core support including phenotype harmonization, data management, sample-identity QC, and

general program coordination were provided by the TOPMed Data Coordinating Center (R01HL-120393; U01HL-120393; contract HHSN268201800001I). We gratefully acknowledge the studies and participants who provided biological samples and data for MESA and TOPMed. JSK was supported by the Pulmonary Fibrosis Foundation Scholars Award and grant K23-HL-150301 from the NHLBI. MRA was supported by grant K23-HL-150280, AJP was supported by grant K23-HL-140199, and AM was supported by R01-HL131565 from the NHLBI. EJB was supported by grant K23-AR-075112 from the National Institute of Arthritis and Musculoskeletal and Skin Diseases.The MESA project is conducted and supported by the National Heart, Lung, and Blood Institute (NHLBI) in collaboration with MESA investigators. Support for MESA is provided by contracts 75N92020D00001, HHSN268201500003I, N01-HC-95159, 75N92020D00005, N01-HC-95160, 75N92020D00002, N01-HC-95161, 75N92020D00003, N01-HC-95162, 75N92020D00006, N01-HC-95163, 75N92020D00004, N01-HC-95164, 75N92020D00007, N01-HC-95165, N01-HC-95166, N01-HC-95167, N01-HC-95168, N01-HC-95169, UL1-TR-000040, UL1-TR-001079, and UL1-TR-001420. Also supported in part by the National Center for Advancing Translational Sciences, CTSI grant UL1TR001881, and the National Institute of Diabetes and Digestive and Kidney Disease Diabetes Research Center (DRC) grant DK063491 to the Southern California Diabetes Endocrinology Research Center

Tiinamaija Tuomi: The Academy of Finland and University of Helsinki: Center of Excellence for Complex Disease Genetics (grant number 312072 and 336826), Folkhalsan Research Foundation, Helsinki University Hospital, Ollqvist Foundation, Liv och Halsa foundation; NovoNordisk Foundation

Teresa Tusie-Luna: CONACyT Project 312688

James S. Ware: Royal Brompton & Harefield Hospitals, Guy’s and St. Thomas’ NHS Foundation Trust, London, UK

Rinse K. Weersma: The Lifelines Biobank initiative has been made possible by subsidy from the Dutch Ministry of Health Welfare and Sport the Dutch Ministry of Economic Affairs the University

Medical Centre Groningen (UMCG the Netherlands) the University of Groningen and the Northern Provinces of the Netherlands

Competing Interests

Mark J. Daly: M.J.D. is a founder of Maze Therapeutics and Neumora Therapeutics, Inc. (f/k/a RBNC Therapeutics).

Mikko Kallela: No related COI

Konrad J. Karczewski: K.J.K. is a consultant for Vor Biopharma, Tome Biosciences, and is on the Scientific Advisory Board of Nurture Genomics.

Eimear E. Kenny: EEK has received personal fees from Regeneron Pharmaceuticals, 23&Me, Allelica, and Illumina; has received research funding from Allelica; and serves on the advisory boards for Encompass Biosciences, Foresite Labs, and Galateo Bio

Mikael Landén: M.L. has received lecture honoraria from Lundbeck pharmaceutical.

Ruth J.F. Loos: R.J.F.L has received consultancy and speaker fees from Novo Nordisk and Eli Lilly and Company

Ronald C.W. Ma: No related COI

Daniel G. MacArthur: D.G.M. is a paid adviser to GlaxoSmithKline, Insitro, and Overtone Therapeutics, and receives research funding from Microsoft Corporation.

Benjamin M. Neale: B.M.N. is a member of the scientific advisory board at Deep Genomics and Neumora.

Anne H. O’Donnell-Luria: A.O.D.L. is on the scientific advisory board for Congenica, receives research funding in the form of reagents from Pacific Biosciences, and is a paid advisor to Addition Therapeutics and former advisor to Tome Biosciences and Ono Pharma USA.

Heidi Rehm: H.L.R. has received rare disease research funding from Microsoft and Illumina and

compensation as a past member of the scientific advisory board of Genome Medical.

Veikko Salomaa: VS has had research collaboration with Bayer Ltd (not related to the present study)

Kaitlin E. Samocha: K.E.S. has received support from Microsoft for work related to rare disease diagnostics

Edwin K. Silverman: Research grants from GSK and Bayer

James S. Ware: JSW has received consultancy fees or grant support from MyoKardia (now Bristol-Myers Squibb), Pfizer, Foresite Labs, Health Lumen, and Tenaya Therapeutics
