# Supplementary material for: Exploring penetrance of clinically relevant variants in over 800,000 humans from the Genome Aggregation Database
Source: Nat Commun. 2025 Oct 31;16:9623. doi: 10.1038/s41467-025-61698-x (PMC12579199; doi:10.1038/s41467-025-61698-x)
Supplement: Supplementary file 2 — Description of Additional Supplementary Files [file 41467_2025_61698_MOESM2_ESM.pdf]

Description of Additional Supplementary Files

Supplementary Data S1: Allele count for allele frequency (AF) bins, Fig. 1d.

Supplementary Data S2: Allele count for P/LP allele count (AC) bins, Fig. 1e.

Supplementary Data S3: Assessment of 90 unique P/LP occurring in combination with pLoF when investigating local pLoF as rescue of 3957 P/LP in ClinVar variants.

Supplementary Data S4: 77 haploinsufficiency genes associated with severe disorders not expected to be compatible with participation in common disease studies (e.g., gnomAD), early onset (before the age of three), and reported as highly penetrant (using *de novo* rate as a proxy for penetrance).

Supplementary Data S5: The specific set of rules used for this project modified for conservative curation using the advanced framework for loss-of-function curation previously published by this group[25].

Supplementary Data S6: Full curation results of 734 pLoF variants found in 77 haploinsufficiency genes in gnomAD v4 genomes.

Supplementary Data S7: Counts for each ClinVar classification and pLoF curation category, 108 of 734 pLoF variants were reported as benign, likely benign, unkown significance, likely pathogenic or pathogenic. Additional 14 variants were reported but excluded due to conflicting interpretations (all 122 variants are included in Figure 3g).

Supplementary Data S8: sQTL results from all pLoF variants.

Supplementary Data S9: Genotypes for sQTL in individuals with MEF2C pLoF variants.
