# Supplementary material for: Exploring penetrance of clinically relevant variants in over 800,000 humans from the Genome Aggregation Database
Source: Nat Commun. 2025 Oct 31;16:9623. doi: 10.1038/s41467-025-61698-x (PMC12579199; doi:10.1038/s41467-025-61698-x)
Supplement: Supplementary file 4 — Reporting Summary [file 41467_2025_61698_MOESM4_ESM.pdf]

nature portfolio

Corresponding author(s): Anne O'Donnell-Luria, Sanna Gudmundsson

Last updated by author(s): Dec 20, 2024

# Reporting Summary

Nature Portfolio wishes to improve the reproducibility of the work that we publish. This form provides structure for consistency and transparency in reporting. For further information on Nature Portfolio policies, see our Editorial Policies and the Editorial Policy Checklist.

## Statistics

For all statistical analyses, confirm that the following items are present in the figure legend, table legend, main text, or Methods section.

| n/a | Confirmed | |
|---|---|---|
| ☐ | ☒ | The exact sample size ($n$) for each experimental group/condition, given as a discrete number and unit of measurement |
| ☒ | ☐ | A statement on whether measurements were taken from distinct samples or whether the same sample was measured repeatedly |
| ☐ | ☒ | The statistical test(s) used AND whether they are one- or two-sided<br>*Only common tests should be described solely by name; describe more complex techniques in the Methods section.* |
| ☒ | ☐ | A description of all covariates tested |
| ☒ | ☐ | A description of any assumptions or corrections, such as tests of normality and adjustment for multiple comparisons |
| ☒ | ☐ | A full description of the statistical parameters including central tendency (e.g. means) or other basic estimates (e.g. regression coefficient) AND variation (e.g. standard deviation) or associated estimates of uncertainty (e.g. confidence intervals) |
| ☐ | ☒ | For null hypothesis testing, the test statistic (e.g. $F$, $t$, $r$) with confidence intervals, effect sizes, degrees of freedom and $P$ value noted<br>*Give P values as exact values whenever suitable.* |
| ☒ | ☐ | For Bayesian analysis, information on the choice of priors and Markov chain Monte Carlo settings |
| ☒ | ☐ | For hierarchical and complex designs, identification of the appropriate level for tests and full reporting of outcomes |
| ☒ | ☐ | Estimates of effect sizes (e.g. Cohen's $d$, Pearson's $r$), indicating how they were calculated |

*Our web collection on statistics for biologists contains articles on many of the points above.*

## Software and code

Policy information about availability of computer code

| | |
|---|---|
| Data collection | No software was used for the collection of data |
| Data analysis | Code used to perform quality control on the gnomAD dataset can be found here:<br>https://github.com/broadinstitute/gnomad_qc<br>bwa-aln<br>Picard version 1.1431<br>VerifyBamID version 1.0.0<br>GATK nightly-2015-07-31-g3c929b0, 3.4-89-ge494930, and 3.6-0-g89b7209<br>VEP version 85 with the LOFTEE plugin (https://github.com/konradjk/loftee)<br><br>Code for generating raw data, ClinVar variants in gnomAD and pLoF variants in gnomAD from publically avalible gnomAD data(D<br>(DOI: 10.5281/zenodo.15175046) |

For manuscripts utilizing custom algorithms or software that are central to the research but not yet described in published literature, software must be made available to editors and reviewers. We strongly encourage code deposition in a community repository (e.g. GitHub). See the Nature Portfolio guidelines for submitting code & software for further information.

## Data

Policy information about availability of data

All manuscripts must include a data availability statement. This statement should provide the following information, where applicable:

- Accession codes, unique identifiers, or web links for publicly available datasets
- A description of any restrictions on data availability
- For clinical datasets or third party data, please ensure that the statement adheres to our policy

The genome aggregation database (gnomAD) v4 dataset can be accessed at https://gnomad.broadinstitute.org/downloads

LoF curation results are avalible in supplementary table and at https://gnomad.broadinstitute.org/

Restrictions: Individual level data required for analysis identifying rescue by local pLoF events in a subset of P/LP in ClinVar, including the finding that a deleterious GJB2 variants is inherited in cis with a LoF variants, is not available to the public.

## Research involving human participants, their data, or biological material

Policy information about studies with human participants or human data. See also policy information about sex, gender (identity/presentation), and sexual orientation and race, ethnicity and racism.

| | |
|---|---|
| Reporting on sex and gender | The gnomAD dataset includes both male and females assigned using genomic data, and thus both sexes are represented in this study. This study investigates all genetic variants of interest regardless sex, thus sex and gender information were not used as part of this work. |
| Reporting on race, ethnicity, or other socially relevant groupings | We investigated all variants associated with disease of interest regardless of race, ethnicity or other socially relevant groupings of the individual. |
| Population characteristics | We did not use additional population characteristic of the samples in most analyses. We report that one genetic finding, a variant rescuing a lethal GJB2-condition, seem to be specific to the East Asian genetic ancestry group. |
| Recruitment | Participants of gnomAD v4 are recruited from over 100 different studies from across the world. A major contributor is the UK Biobank consisting of approx. 500,000 exomes from individuals recruited in the UK. For most other studies, individuals were recruited as part of studying a certain, mostly complex, disorders like heart disease or diabetes. This is a heterogeneous cohort of individuals, we have a mix of genetic ancestry groups. |
| Ethics oversight | Broad Institute of MIT and Harvard, Mass General Brigham IRB |

Note that full information on the approval of the study protocol must also be provided in the manuscript.

# Field-specific reporting

Please select the one below that is the best fit for your research. If you are not sure, read the appropriate sections before making your selection.

☒ Life sciences ☐ Behavioural & social sciences ☐ Ecological, evolutionary & environmental sciences

For a reference copy of the document with all sections, see nature.com/documents/nr-reporting-summary-flat.pdf

# Life sciences study design

All studies must disclose on these points even when the disclosure is negative.

| | |
|---|---|
| Sample size | We used all individuals in the full Genome Aggregation Database (gnomAD) v4 dataset, which includes 807,162 individuals. For the pLoF analysis we used the subset of individuals that had genome sequence data: 76,215 individuals. |
| Data exclusions | No data was excluded from the gnomAD v4 dataset. The quality control pipeline defining samples and variants included in the final gnomAD set is described here https://gnomad.broadinstitute.org/news/2023-11-gnomad-v4-0/ |
| Replication | All results can be replicated using publically avalible data (https://gnomad.broadinstitute.org/downloads) and code that filters all ClinVar and all pLoF variants in the 77 genes of interest in gnomAD v4 (DOI: 10.5281/zenodo.15175046). The analysis of local pLoF rescue of potential gain of function variants identifying one example in GJB2 requires individual level data and thus cannot be replicated. |
| Randomization | This was a population-based study, not a case-control study, so no randomization was performed. |
| Blinding | This was a population-based exploratory study and blinding does not apply. |

# Reporting for specific materials, systems and methods

We require information from authors about some types of materials, experimental systems and methods used in many studies. Here, indicate whether each material, system or method listed is relevant to your study. If you are not sure if a list item applies to your research, read the appropriate section before selecting a response.

## Materials & experimental systems

| n/a | Involved in the study |
| --- | --- |
| ☒ | ☐ Antibodies |
| ☒ | ☐ Eukaryotic cell lines |
| ☒ | ☐ Palaeontology and archaeology |
| ☒ | ☐ Animals and other organisms |
| ☒ | ☐ Clinical data |
| ☒ | ☐ Dual use research of concern |
| ☒ | ☐ Plants |

## Methods

| n/a | Involved in the study |
| --- | --- |
| ☒ | ☐ ChIP-seq |
| ☒ | ☐ Flow cytometry |
| ☒ | ☐ MRI-based neuroimaging |

## Plants

| | |
| --- | --- |
| Seed stocks | N/A |
| Novel plant genotypes | N/A |
| Authentication | N/A |
